# Traditional Medicine and Its Role in the Management of Diabetes Mellitus: “Patients' and Herbalists' Perspectives”

**DOI:** 10.1155/2019/2835691

**Published:** 2019-07-04

**Authors:** Rose Kasole, Haikael D. Martin, Judith Kimiywe

**Affiliations:** ^1^Department of Food Biotechnology and Nutritional Sciences, The Nelson Mandela African Institution of Science and Technology (NM-AIST), Arusha, Tanzania; ^2^Department of Economic and Productive Sector, Iringa Regional Secretariat (RAS), P.O. Box 858, Iringa, Tanzania; ^3^Department of Food, Nutrition and Dietetics, Kenyatta University (KU), Nairobi, Kenya

## Abstract

**Background:**

Diabetes mellitus is a complicated health condition with multiple causes and many treatment options. Various myths may influence diabetics' health-seeking behavior, and they may use traditional medicines, which include normal foods and herbs, for primary health care. The aim of this study was to determine patients' and herbalists' practices and perspectives regarding the use of traditional medicines and the role of traditional medicines in the management of diabetes.

**Methods and Findings:**

We conducted a cross-sectional study with a mixed-methods design. We interviewed 140 patients attending diabetic clinics using a structured questionnaire, conducted focus group discussions with an additional 20 diabetic patients, and conducted in-depth interviews with 8 local herbalists. The majority of the diabetic participants believed that diabetes is caused by a high-carbohydrate diet. Of the 140 participants who answered the questionnaire, 67.2% reported using traditional medicines to manage their diabetes, including 58.6% who reported using both conventional medicines and traditional medicines. Some participants believed that combining conventional and traditional medicines improved the effectiveness of treatment. Reasons given for using traditional medicines included the high cost of conventional treatment and the availability and accessibility of the traditional medicines. The most commonly used traditional medicines were indigenous vegetables and medicinal plant products including amalanth leaves, hare lettuce leaves, nightshade leaves, spider plant leaves, okra pods, moringa leaves and seeds, soursop leaves, black plum back, avocado seed, and lemongrass.

**Conclusion:**

Patients and herbalists provided a range of perspectives regarding the use of traditional medicines to treat diabetes. Further research is needed to identify bioactive compounds present in commonly used traditional medicines and their efficacy.

## 1. Introduction

Diabetes mellitus is a common metabolic health problem. Globally, the prevalence of diabetes in adults has risen rapidly from 4.7% in 1980 to 8.5% in 2014 [1]. Hyperglycemia is one of the features of diabetes [2]. Hyperglycemia increases the risk of diabetes and associated complications including heart attack, stroke, kidney disease, limb amputations, poor vision, and nerve damage [1, 3]. Diabetes and its complications are leading causes of death worldwide, the increased percentage of death worldwide [1].

It has been estimated that in Africans about 80% of cases of diabetes are undiagnosed. This may be because diabetes is often asymptomatic or produces only mild symptoms, which may be ignored or attributed to other causes [2, 4]. Many people believe in spiritual and alternative treatments and use these treatments in preference to seeking care from medical doctors [5]. Many people also believe in traditional ways of living, and this influences their health-seeking behavior. Traditional medicines, which include normal foods and herbs, are used as the main form of primary health care.

Studies reported that 80% of people in developing countries depend on traditional medicines as the primary remedy for various ailments [6, 7]. Worldwide, plant-based traditional medicines are the most commonly used form of treatment for a range of health problems. These traditional medicines play an important role in primary health care in many developing countries. Tanzania is among the countries where the majority of the population depends on traditional medicines for the management of their health problems, including diabetes [8]. Lunyera et al. [9] reported that 77% of diabetic patients in Northern Tanzania use traditional medicines for the management of diabetes. Maregesi [10] reported that there are various traditional medicines used by diabetic patients in Northern Tanzania, many of which have not been documented, and their pharmacological properties with respect to blood glucose control have not been studied. The use of traditional medicines may be attributable to sociocultural perspectives within the community, and they may contribute to disease-related complications. Most traditional medicines are common foods which include vegetables, flowers, fruits, seeds, spices, and herbs. Some foods provide health benefits beyond their nutritional value, and are effective in the prevention and treatment of various diseases [11, 12]. The objective of this study was to describe patients' and herbalists' perspectives and practices regarding the use of traditional medicines and the role of traditional medicines in the management of diabetes in Northern Tanzania.

## 2. Materials and Methods

### 2.1. Study Design

We conducted a cross-sectional study with a mixed-methods design which included a quantitative and a qualitative component. The methods were complementary and enabled triangulation of the data [13]. Focus group discussions (FGDs) and in-depth interviews (IDIs) were used to gather qualitative data on patients' and herbalists' perspectives on the use of traditional medicines in the management of diabetes mellitus. A structured questionnaire was used to gather quantitative data from the patients to supplement the data provided by participants in the FGDs.

### 2.2. Study Setting

The study was conducted in Kilimanjaro Christian Medical Centre (KCMC) in Kilimanjaro, and Mount Meru Hospital in Arusha. These facilities provide specialized diabetes care in Northern Tanzania. The Kilimanjaro Region has a population of 1.64 million, and the Arusha Region has a population of 1.69 million [14]. KCMC is a zonal referral hospital located on the slopes of Mount Kilimanjaro, established by the Good Samaritan Foundation (GSF) in 1971. Mount Meru Hospital is a government-owned regional referral hospital located in the foothills of Mount Meru in Arusha municipality, established in 1926.

### 2.3. Study Population

The study participants were diabetic outpatients and local herbalists or traditional medicine vendors. Participants were required to be older than 20 years in order to ensure that they were able to provide reliable information. Pregnant women were excluded because of the possibility that they may have had gestational diabetes, which resolves after giving birth.

### 2.4. Sample Size Determination and Sampling Techniques

Purposive sampling was used to select hospitals, patients and herbalists [15]. The patients were stratified according to their sex; two strata of 10 patients each were formed, in which FGDs and questionnaire participants were randomly drawn by counting numbers and selecting all even numbers to include in the interview. There were 4 FGDs with 5 participants in each. One FGD for males and one FGD for females were conducted at each hospital. Snowball sampling was used to recruit herbalists to participate in in-depth interview (IDI); 4 potential participants were picked from each region (Kilimanjaro and Arusha) at their working place/shops for interview. The sample for questionnaire participants was obtained by using a formula adapted from Gelaw, Abdela [4], with the following parameters: 9.1% prevalence of diabetes (P), 5% margin of error (E), and a standard normal deviation (Z) of 1.96, and a contingency of 10%.

(1) Formula;  N=Z2P1−PE2+10%  contingencyN=1.9620.0911−0.0910.052=127,10%  contingency  of  127=12710100=12.7127+12.7=139.7≈140

The target sample size was 140 patients to answer the questionnaire, 20 patients for FGDs, and 8 herbalists for IDIs.

### 2.5. Data Collection

Quantitative data were collected using a questionnaire which contained both closed- and open-ended questions. This questionnaire collected sociodemographic information and contained questions on treatment choices, use of traditional medicines, the reasons for choosing to use traditional medicines, the source of the medicines, the diabetes-associated complications for which each medicine was used, and the effectiveness of the medicines. The questionnaire was developed in English, translated into Swahili, pretested on 8 diabetic patients from KCMC prior to starting data collection, and revised based on their responses. The questionnaire was administered by an interviewer.

FGDs and IDIs were conducted with patients and herbalists, respectively, to gather information on their perceptions and practices regarding the use of traditional medicines in the management of diabetes. The discussions and interviews were audio-recorded and a research assistant took notes during each interview. This material was transcribed and translated into English by one of the researchers (RK) and was then reviewed and moderated by a qualitative data analyst expert to ensure accuracy of the translations.

### 2.6. Data Analysis

Quantitative data were analyzed using SPSS software, version 20. After the data had been entered, a descriptive analysis was done, and the results were reported as frequencies and percentages. Qualitative data were analyzed according to the method described by Miles and Huberman, which entailed highlighting, summarizing, or discarding different sections of the text [16]. The data were then organized, synthesized, and interpreted based on the themes of the study.

### 2.7. Ethical Considerations

The National Institute for Medical Research (NIMR) provided ethical approval, and hospital authorities provided permission to conduct the study in the two health facilities. Participants provided a written informed consent to participate in the research. The findings from the study will be used to recommend for better services. There were no known risks for the participants; interviews were conducted in a comfortable environment.

## 3. Results

### 3.1. Sociodemographic Characteristics

Table 1 shows the sociodemographic characteristics of 140 participants who answered the questionnaire. Of these participants, 66.4% were female, 50.7% were aged between 41 and 60 years, 55.7% were from KCMC, 63% had a primary level of education, 48.6% were self-employed, and 64.2% had had diabetes for more than five years.

There were 4 FGDs with 5 diabetic outpatients in each. One FGD for males and one FGD for females were conducted at each hospital. Also, 8 herbalists (4 from each region) who sold their herbs in a market at their shops participated in the IDI.

### 3.2. Patients' and Herbalists' Perspectives toward Diabetes

The perspectives on diabetes reported by the participants who answered the questionnaire are shown in Table 2. Some participants reported that diabetes is a disease like any other disease. The majority of participants were aware that diabetes can be caused by a high-carbohydrate diet and that diabetes is treatable. The majority of participants reported that they preferred to use a combination of conventional medicines and traditional medicines to treat their diabetes, and only a small minority of participants reported that they preferred to use traditional medicines alone.

The perspectives expressed by the FGD and IDI participants were similar to those of the participants who participated in the quantitative component of the study. Regarding the causes of diabetes, some participants thought that diabetes is an inherited disease; some thought that it is a result of failure of organs such as the pancreas; and others thought that it was related to dietary factors such as a high intake of carbohydrates, sugar, sweets, alcohol, or fatty foods. The majority of participants believed that diabetes occurs as a result of a high-carbohydrate diet. Most participants believed that diabetes could be cured, and most of the participants in the FGDs participants reported that they thought that using both conventional medicines and traditional medicines was more effective than using either type of medicine alone. These findings are summarized in Table 3.

From the data collected during the IDIs and FGDs, it was evident that participants believed that the type of food eaten and eating behavior could contribute to the development of diabetes. The following quotes provide examples of participants' perspectives on diabetes:

“A high intake of carbohydrates contributes to the disease epidemic, because most of the foods that we eat in town are rich in carbohydrates compared to those eaten in the village, where they eat traditional foods.” (*FGD*  *Participant*  1, 05/18/2018)

“…I'm using conventional and traditional medicines at different times, for example, if I use conventional medicines at around 6 o'clock in the morning, I wait at least 2 hours, until about 8 o'clock, before taking traditional medicines. We do this in order to cure the disease.” (*FGD*  *Participant*  11, 05/20/2018)

### 3.3. Traditional Medicines, Sources, and Practices among Diabetic Patients

**Figure 1:**
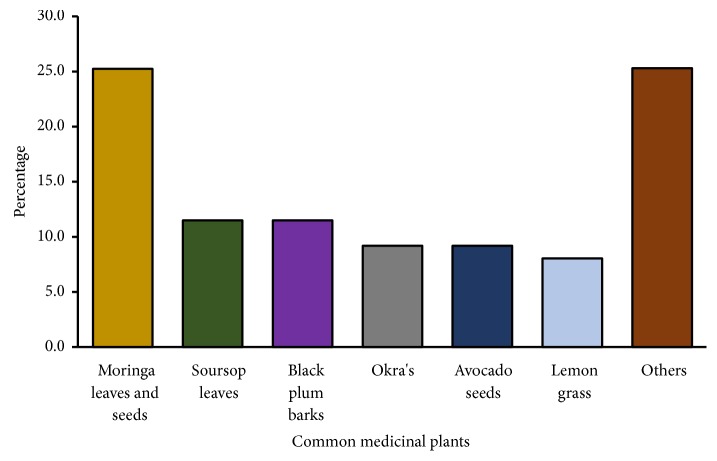
Popular medicinal plants for diabetes reported by the participants who answered the questionnaire.

Figure 1 shows the most common traditional medicines that participants who answered the questionnaire reported that they used in the management of diabetes. Moringa leaves and seeds (*Moringa oleifera*) (25.2%), soursop leaves (*Annona muricata*) (11.5%), black plum bark (*Syzygium cumini*) (11.5%), okra pods (*Abelmoschus esculentus*) (9.2%), avocado seeds (*Persea americana*) (9.2%), and lemongrass (*Cymbopogon citratus*) (8%) were the traditional medicines reported most frequently, and 25.3% of participants reported using other traditional medicines.

Table 4 shows the sources used to obtain traditional medicines among participants who answered the questionnaire and their practices regarding the use of traditional medicines for managing diabetes.

Participants in the FGDs and IDIs had similar perceptions to participants in the quantitative component of the study regarding foods that can be used to treat diabetes. The majority identified vegetables such as okra pods, local varieties of green leafy vegetables such as amaranth leaves, spider plant, hare lettuce and nightshade leaves, and spices such as ginger, garlic, and cinnamon as remedies for diabetes and reported that eating large quantities of these vegetables helps to treat diabetes. In addition, they identified several types of herbs and herbal preparations which they believed to be useful in curing diabetes. These included* Aloe vera*, moringa leaves and seeds, black plum bark, avocado seeds, rosemary flowers (*Vinca rosea*), bitter melon, lemongrass, and soursop leaves. In most cases, these medicines were mixed with warm water, milk, tea, or porridge or drunk neat if the medicine was in liquid form. Most participants stated that the duration of using traditional medicines depended on their health status.

Most of the traditional medicines were available locally. The majority of participants in the FGDs reported that they obtained most of their traditional medicines from markets, neighbors, or traditional medicine vendors, and some participants reported that they had planted medicinal plants around their homes. The local herbalists reported that they obtained most of the medicines that they used to treat diabetes locally and that they prepared them themselves, based on their experience.  Table 5 summarizes the key findings regarding the types of medicinal plants and traditional medicines that participants in the FGDs and IDIs used and their source.

From the data collected during the IDIs and FGDs, it was evident that moringa leaves and seeds, okra pods, black plum bark, soursop leaves, lemongrass, avocado seed, and indigenous vegetables such as nightshade leaves, spider plant leaves, amaranth leaves, and hare lettuce leaves were popular for treating diabetes. The following quotes provide examples of participants' comments regarding their use of medicinal plants and traditional medicines:

“I'm using moringa seeds and rosemary flowers as remedies for my diabetes. These have been very helpful and, since I started using them, my blood sugar level has dropped to normal.” (*FGD*  *Participant*  15, 05/20/2018)

“We obtain them [traditional medicines] from herbal medicine shops in Arusha, and collect others from farms and forests. I buy fruit and vegetables such as carrot, garlic, ginger, onions, and cinnamon, from the market. I buy shubiri [dried Aloe vera] from the market because I cannot process it.” (*Local*  *Herbalist*  *E*, 6/23/2018)

### 3.4. Reasons for Using Traditional Medicines

The main reasons that participants who answered the questionnaire gave for using traditional medicines were the high cost of conventional medicines, availability and accessibility of traditional medicines, and advice from friends (Table 6).

The reasons for using traditional medicines among FGD participants were similar to those given by participants who answered the questionnaire. They also reported that the cost of conventional medicine, the accessibility and availability of the traditional medicines, and advice from friends led them to use traditional medicines. Some participants believed that using a combination of conventional and traditional medicines was more effective than using either type of medicine alone. In addition to diabetes, some participants believed that traditional medicines were effective for managing other conditions associated with diabetes, such as slow wound healing and kidney problems, along with relief from constipation and high blood pressure. Moringa seeds were said to be useful for managing high blood pressure and for healing wounds. However, the majority of participants were aware that traditional medicines can have adverse effects such as hypoglycemia, kidney disease, fatigue, and diarrhea.

Some of the local herbalists reported that traditional medicines have varying effectiveness. They attributed this to unclear prescriptions on how much to take or how long to take it for. Additionally, some participants in the FGDs and IDIs expressed the opinion that conventional medicines were not as helpful as traditional medicines because they brought relief but did not provide a cure. Furthermore, some FGD respondents stated that the government should certify and support the use of traditional medicines because they are effective at treating many diseases that cannot be treated in hospitals. Herbalists agreed that the government should support the use of traditional medicines and gave examples of people who had not been treated in a hospital but had been treated successfully with locally made medicines (Table 7).

From the summary presented in Table 7, it is apparent that most of the participants preferred to use traditional medicines rather than conventional medicines; even though they thought that most traditional medicines are very slow to take effect. Also, some participants expressed concern that some traditional medicines may have adverse effects due to a lack of specifications and standards on how much to use. The following quotes provide examples on reasons for using, or not using, traditional medicines:

“Sometimes I do not go to the hospital to collect my medicine because I do not have enough. I then use traditional medicines because they are available and accessible.” (*FGD*  *Participant*  4, 05/18/2018).

“Soursop leaves are good for managing diabetes, but one has to drink only very little because if you drink too much, your blood sugar drops and becomes extremely low. One day I used it and I immediately experienced side effects”. (*FGD*  *Participant*  7, 05/20/2018)

“People are interested in using medicines for a short period of time. When patients realize that they have to use a medicine for a long time, such as 90 days, they get bored. Traditional medicines work slowly and they need to be taken in the correct dose for the required amount of time.” (*Local*  *Herbalist*  *F*, 06/24/2018)

### 3.5. Association between Sociodemographic Characteristics and Choice of Treatment

Table 8 shows the association between sociodemographic characteristics and choice of treatments among the participants who answered a questionnaire. The only characteristic that appeared to influence their choice was age, with people aged 41–60 years being more inclined to use a combination of conventional and traditional medicines. In all age groups, only a small minority chose to use traditional medicines alone.

## 4. Discussion

Diabetes is a complicated health condition with multiple risk factors and many ways of management. It is accompanied by various myths and misconceptions. Myths may exist because of a lack of knowledge and awareness of diabetes and close-mindedness. In order to overcome this, there is a need to study community beliefs and perceptions toward health-seeking behaviors. This study focused on the patients' and herbalists' perspectives and practices regarding the use of traditional medicine and its role in the management of type 2 diabetes mellitus. We found that participants have a range of beliefs regarding the causes and treatment of diabetes.

Regarding the cause of diabetes, the majority of the study participants perceived that diabetes occurs as a result of a high-carbohydrate diet. This finding is similar to that of Rai and Kishore [5], who found that participants considered sugar to be the cause of diabetes. Some of the participants in this study believed that rice raises blood glucose levels because of its high-carbohydrate content and that rinsing it several times or soaking it in water can remove carbohydrates. This misconception is similar to that reported on another study, in which participants stated that carbohydrates should be removed from diabetic diets [17]. In fact, people need carbohydrates as a major source of energy for the body's cells and brain [18–21]. Due to that, it is impossible to totally avoid carbohydrate source foods, but the choice of carbohydrate source foods to consume is important for the prevention of diabetes. Overconsumption of simple carbohydrates, such as fructose and glucose, increases lipid deposition in the liver and muscles and reduces insulin sensitivity [22, 23]. Most refined grains are rich in simple carbohydrates which are absorbed rapidly, cause the blood glucose level to rise soon after the meal, and contain insufficient amounts of other nutrients such as fiber, proteins, vitamins, and minerals [24, 25]. Rinsing foods such as rice several times or soaking foods in water does not remove carbohydrates but may deplete the food of important nutrients such as water-soluble vitamins, minerals, and fiber [26–28]. Consumption of whole grain as a source of carbohydrates is preferable to consuming refined grains because of lower glycemic responses that help the dietary management of diabetes and hyperglycemia [29, 30]. Therefore, awareness needs to be created of which carbohydrates are bad for one's health and their importance to the body's cells and organs.

Also, many of the study participants believed that there are foods and traditional medicines that can be used to treat diabetes. This finding is similar to the findings of other studies [17, 31, 32] which have identified various indigenous vegetables, spices, and herbs that may be useful in treating diabetes. In our study, the vegetables and spices that were used by diabetic patients and local herbalists in the management of diabetes included locally grown amaranth leaves (*Amaranth species*), hare lettuce (*Sonchus luxurians*), nightshade leaves (*Solanum villosum millers*), spider plant leaves (*Gynandropsis gynandra*), and okra pods (*Abelmoschus esculentus*). Traditional medicines which were found to be widely used included moringa seeds and leaves (*Moringa oleifera*), soursop leaves (*Annona muricata*), black plum tree bark (*Syzygium cumini*), avocado seeds* (Persea americana*), lemongrass (*Cymbopogon citratus*),* Aloe vera*, and rosemary flowers (*Vinca rosea*). These traditional medicines are usually used as food or food ingredients and some are used as herbs in some countries, including Tanzania. Most of these foods and traditional medicines used on diabetes management contain antioxidants, phytochemicals, polyphenols, vitamins, and minerals which provide scavenging effects toward oxidative stress [33, 34].

The vegetables and traditional medicines identified by participants in our study have been reported in other studies as medicinal plants that may be used to treat various ailments, including diabetes [7, 9, 35–38]. Two-thirds of the diabetic patients in this study reported that they used vegetables and traditional medicines as remedies for diabetes and related conditions such as high blood pressure, wounds, eyes, and kidney problems. Secondary metabolites and phytochemicals in some medicinal plants act as antioxidants and prevent the development of chronic diseases such as diabetes [39–41].

In addition, in this study, the effectiveness, availability, and accessibility of traditional medicines; the cost of conventional medicines; and the advice of friends and relatives were driving forces for using traditional medicines. These findings are similar to those reported in other studies [9, 42, 43]. One study found patients who were persuaded to use herbal medicines because of a free choice of service providers and encouragement by friends and relatives [44]. However, limited knowledge of the potential adverse effects, effective dose, and rational use is still a challenge, notwithstanding the promising results of some medicinal plants and traditional medicines on disease control, management, and treatment [45].

The safety of the traditional medicines is a big concern among people and global health authorities, because of their use by unlicensed or uncertified practitioners, as well as self-medication [46, 47]. In our study, some participants reported that when they fell ill, they would find traditional medicines in the bushes around their home or would acquire traditional medicines from local herbalists, friends, and relatives. Some of the participants in the FGDs and IDIs reported that they considered traditional medicines to be safe because they have no chemicals. Other studies have also discovered that participants considered traditional medicines to have no chemicals [48, 49]. However, plants contain natural chemical substances, some of which are toxic [50]. In our study, unclear specifications/standards and toxicity of the traditional medicines were some of the factors that made some participants to consider traditional medicines as unsafe. They believed that traditional medicines could damage organs such as the kidneys and the liver and could lead to other complications, including hypoglycemia, diarrhea, joint pains, and muscular weakness. There have been reports on the adverse effects of some herbal medicines. The adverse effects of herbal medicines can be life-threatening and can cause of premature death [6, 51, 52]. Hussin [48] reported on the frequency of adverse effects associated with the use of traditional medicine in Malaysia and attributed these adverse effects to the presence of heavy metals and the multiple active ingredients found in the traditional medicines.

Bush and Rayburn [53] and Armstrong and Thiébaut [54] reported on the use of both conventional and traditional medicines among their participants. Their findings were similar to our study results. They found that the majority of participants used both traditional and conventional medicines and that fewer participants used traditional medicines alone. This could be attributed to the belief that conventional medicines and traditional medicines have a complementary effect and that using both medicines is more effective. However, some participants reported that they used traditional medicines to offset the cost of conventional medicines and as a back-up in the event that they were unable to access conventional medicines due to income constraints. However, use of herbal/traditional medicines in combination with conventional medicine may compromise the safety and efficacy of treatment due to drug interactions [55–57]. These interactions may lead to adverse effects such as those reported by some of the participants, which may lead to coma and premature death. Bush and Rayburn [53], reported on the hypoglycemic effect of herb-drug interactions among patients who took the traditional medicine, Nepal, with metformin or glyburide, and hypertension caused by herb-drug interactions among patients who took ginkgo with hydrochlorothiazide. It is thus important to create awareness in the community of the potential harmful effects and the potential benefits of using traditional medicines.

This study found that participants' age influenced their choice of treatment for managing their diabetes and that as age increased participants were more likely to use traditional medicines. This finding verified by the absolute percentages where 76% (54/71) of participants aged 40-60 years, 58.2% (32/55) of the participants aged >60 years, and 57% (8/14) of those aged 20-40 years used traditional medicines alone or in combination with conventional medicine. Other studies have also found that older adults were more likely to use complementary and alternative medicines [54, 58]. This could be because older adults have accumulated enough knowledge of the varieties and health benefits of traditional medicines. However, other studies have found that the majority of users of alternative medicines are young or middle aged [59–61]. Our study did not find an association between sex, marital status, employment status, income, or the duration of diabetes and participants' treatment choices. Similarly, some other studies have not found an association between use of traditional medicines and the sociodemographic characteristics of participants [17, 62].

This study has some limitations. Apart from the 8 traditional herbalists, all the participants had diabetes and were attending diabetic clinics at the specialized referral hospitals, and the study did not include diabetic patients attending primary health care facilities. Due to this, the findings might not be representative of all diabetic patients in Northern Tanzania. Also, the results may be biased because of the sex imbalance with fewer male participants than female patients (33.6% versus 66.4%). This sex imbalance is due to the relatively small number of men attending the selected diabetic clinics. Social desirability bias was also observed because participants were recruited in a medical setting, and the researchers had a biomedical background; this may have influenced participants to under-report their use of traditional medicines and to respond to questions in a manner that they thought the researchers would approve of. Also, apart from not including diabetics attending primary care facilities, the study did not include diabetics who were not in care. Diabetics who are not in care are likely to make even greater use of traditional medicines and by definition; they do not use conventional medicines.

## 5. Conclusion

This study assessed patients' and herbalists' perspectives and practices regarding the use of medicinal plants and traditional medicines in the management of diabetes. The majority of participants used medicinal plants and traditional medicines in the management of their diabetes, either alone or in combination with conventional medicines. Most of the traditional medicines were plant products that were affordable and widely available locally. Although traditional medicines have potential benefits, they also have potential adverse effects. There is currently limited knowledge of the physiological effects of most of the traditional medicines that were widely used, so it is not possible to assess whether these medicines were beneficial, harmful, or both. In view of this, the findings reveal a need for further research to identify the bioactive compounds present in these medicinal plants and to determine their efficacy at a physiological level.

## Figures and Tables

**Table 1:** Sociodemographic characteristics of the participants in the quantitative component of the study (N=140).

Characteristics	n	%
*Gender*		
Male	47	33.6
Female	93	66.4
*Age (years)*		
21-40	14	10.0
41-60	71	50.7
>60	55	39.3
*Clinic center*		
KCMC	78	55.7
Mount Meru	62	44.3
*Marital status*		
Never married	10	7.1
Married or cohabiting	102	72.9
Separated or divorced	6	4.3
Widowed	22	15.7
*Level of education*		
Never went to school	5	3.6
Primary school	89	63.6
Secondary school	23	16.4
College/ university	23	16.4
*Employment status*		
Formal employment	24	17.1
Self-employed	68	48.6
Retired officer	19	13.6
Unemployed	29	20.7
*Duration of the disease*		
<1 Year	7	13.2
1-5 years	12	22.6
>5 years	34	64.2

*N*: sample size; *n*: number of participants.

**Table 2:** Participants' perceptions in the quantitative component of the study (N=140).

Variable	n	%
*Cause of diabetes*		
Disease due to unhealthy eating	39	27.9
Inherited and life style	19	13.5
Disease like other diseases	54	38.6
Disease due to stress	12	8.6
I don't know	16	11.4
*Food cause diabetes*		
Carbohydrate rich foods	83	59.3
Red meat and fats	11	7.9
Alcohol and soft drinks	11	7.9
Sugar and sweets	8	5.7
No foods that cause diabetes	27	19.3
*Is diabetes treatable?*		
Yes	83	59.3
No	57	40.7
*Treatment choice*		
Conventional	46	32.9
Traditional	12	8.6
Conventional and traditional	82	58.6

*Note*. n: number and %: percent.

**Table 3:** Summary of perspectives on diabetes provided by participants in the focus group discussions and in-depth interviews.

Theme	Data source	Participants' Key message	Participants' remark
Perception and understanding of diabetes	FGDs and IDIs	This is a disease caused by lifestyle, particularly unhealthy eating habits.	Lifestyle affects one's personal health in a variety of ways.

Foods that cause diabetes	FGDs and IDIs	Diabetes is caused by a high intake of carbohydrates such as sugar, sweets, rice, potatoes, alcohol, Coke. Foods grown with fertilizer also cause diabetes.	A diet that contains a lot of vegetables can help to keep diabetes under control.
			Some participants believed that drinking lots of water can overcome the adverse effects of carbohydrates and chemicals or toxins in the diet.
		Rice raises blood glucose levels because of its high carbohydrate content.	Rinsing rice several times, or soaking it in water, can remove carbohydrates.

Preferred type(s) of treatment	FGDs	Medicines prescribed by a physician and traditional medicines can be used interchangeably.	Most diabetic participants were looking for a treat of diabetes, and believed that the use of conventional medicines and traditional medicines is the most effective option.

FGD, focus group discussion; IDI, in-depth interview.

**Table 4:** Sources of traditional medicines and practices regarding the use of traditional medicines among participants who answered the questionnaire (N=140).

Questions and responses	n	%
*Source of traditional medicine*		
Traditional medicine vendors/herbalists	44	31.4
Other patients, relatives and friends	50	35.7
Did not use traditional medicine	46	32.9
*Traditional medicine preparation*		
Used in raw form by chewing or making as juice	16	11.4
Dried, blended and mixed with milk or tea	30	21.4
Boiled or soaked in water, and decanted the liquid	48	34.3
Did not use traditional medicine	46	32.9
*Frequency of using traditional medicines*		
Twice per day	40	28.6
Three or more times per day	41	29.3
When their blood glucose level rises	13	9.3
Did not use traditional medicine	46	32.9

**Table 5:** Summary of responses given by participants in the focus group discussions and in-depth interviews, regarding the types of traditional medicines that they used and their source.

Theme	Data source	Participants' Key message	Participants' remark
Foods used to treat diabetes	FGDs and IDIs	Intake of large quantity of indigenous vegetables such as amaranth leaves, hare lettuce, spider plant leaves, nightshade leaves, okra pods, African eggplant, ginger, garlic, cinnamon and tamarind juice	These vegetables are generally eaten as part of the meal. Some medicinal plants, especially okra pods, are used in medicinal doses.

Common traditional medicines/herbs used to treat diabetes	FGDs and IDIs	Moringa seeds and leaves, rosemary flowers (*Vinca rosea*), bitter melon, soursop leaves, avocado seeds black plum bark, lemongrass, and *Aloe vera* were the most common plants that were used to treat diabetes	Patients reported that some of the plants are effective in managing diabetes and other related health conditions if used properly, but some are not.

Sources of traditional medicines	IDIs	Herbalists obtained most of the plants that they used for preparing traditional medicines, from forests and farms	Generally, plants used for traditional drug preparation are locally available
	FGDs	Diabetic patients obtained from of their traditional medicines from their homes, neighbors, friends, and markets	

Abbreviations: FGD, focus group discussion; IDI, in-depth interview.

**Table 6:** Reasons for using traditional medicines for diabetes given by participants who answered a questionnaire (N=140).

Variables	n	%
*Reason for deciding to use traditional medicine*		
High cost of the conventional medicines	25	17.9
Accessibility and availability of traditional medicine	24	17.1
Advice of friends and relatives	45	32.1
Do not use traditional medicine	46	32.9
*Usefulness of the medicine on managing diabetes*		
Leads to very low blood pressure and hypoglycemia	17	12.1
Regulate blood glucose levels	77	55
Do not use traditional medicine	46	32.9
*Reason for not using traditional medicines*		
Religious beliefs	3	9.3
Advice from health care providers	13	9.3
Not aware of the effectiveness or how to use it	11	7.9
Prefer not to mix treatment with conventional medicines and traditional medicines	9	6.4
Do use traditional medicine	94	67.1
*Reasons for using both conventional medicines and traditional medicines*		
More effective	28	20
Cost substitution	16	11.4
Looking for effective treatment	27	19.3
Not applicable	69	53.6
*Complications of using traditional medicines*		
High blood pressure	42	30
Eye, kidney, and joint problems	18	12.9
Diabetes only	34	24.3
Do not use traditional medicines	46	32.9

**Table 7:** Summary of reasons for using traditional medicines provided by participants in the focus group discussions and the in-depth interviews.

Theme	Data source	Participants' Key message	Participants' remark
Influence and source of information on the use of traditional medicines for managing diabetes	FGDs	Conventional medicines are costly Traditional medicines are readily available and accessible Choice of medicine type influenced by friends, TV and radio advertisements, traditional medicine vendors/herbalists	If possible, medicines for diabetes should be provided for free, because some patients do not seek treatment due to a lack of money. Community-based counseling can raise awareness of diabetes and knowledge regarding its management.

Effectiveness of traditional medicines and conventional medicines	IDIs	The effectiveness of any medicine depends on the duration of the disease.	Any type of drug performs better in the early stages of the disease.
		Traditional medicines do not work as fast as conventional medicines.	Traditional medicines have a gradual effect and people should complete their course of treatment.
	FGDs	Traditional medicines help in the management of complications of diabetes	Traditional medicines help to manage high blood pressure, eye and kidney diseases, joint pains, and to heal wounds, among others.

Challenges and side effects associated with the use of traditional medicines	FGDs	Traditional medicines, if not used appropriately can have adverse effects such as diarrhea, fatigue, extreme decrease blood sugar level, and can sometimes lead to death	It is preferable for diabetes to be treated in hospitals using controlled doses of conventional medicines.
		Traditional medicines have varying effectiveness	
		It is not clear how much traditional medicine to take, or how long to take it for.	Research should be done in order to determine the most effective dose

Recommendations/ suggestions	FGDs and IDIs	The government should recognize, certify and support the use of traditional medicines	To follow-up with the traditional medicine vendors/herbalists

FGD, focus group discussion; IDI, in-depth interview.

**Table 8:** Sociodemographic characteristics of participants in the quantitative component of the study in relation to their preferred type of treatment.

Variables	Treatment choice			
	Conventional	Traditional	Conventional & traditional	P-Value
*Gender*				
Male	17(37.0)	4(33.3)	26(31.7)	0.83
Female	29(63.0)	8(66.7)	56(68.3)	

*Age (years)*				
21-40	6(13.0)	3(25.0)	5(6.1)	0.05
41-60	17(37.0)	5(41.7)	49(59.8)	
>60	23(50.0)	4(33.3)	28(34.1)	

*Marital status*				
Never married	6(13.0)	0	4(4.9)	0.43
Married or cohabiting	30(65.2)	8(66.7)	64(78.0)	
Separated or divorced	2(4.3)	1(8.3)	3(3.7)	
Widowed	8(17.4)	3(25.0)	11(13.4)	

*Education*				
Never went to school	0	1(8.3)	4(4.9)	0.61
Primary school	29(63.0)	9(75.0)	52(63.4)	
Secondary school	9(19.6)	1(8.3)	12(14.6)	
College/ university	8(17.4)	1(8.3)	14(17.1)	

*Employment status*				
Formal employment	9(19.6)	1(8.3)	14(17.1)	0.79
Self-employed	19(41.3)	7(58.3)	44(53.7)	
Retired officer	10(21.7)	3(25.0)	14(17.1)	
Unemployed	8(17.4)	1(8.3)	10(12.2)	

*Duration of the disease*				
<1 Year	4(8.7)	0	4(4.9)	0.76
1-5 years	10(21.7)	3(25.0)	22(26.8)	
>5 years	32(69.6)	9(75.0.0)	56(68.3)	

Chi-square test (95% CI, p<0.05).

## Data Availability

The data used to support the findings of this study are available from the corresponding author upon request.
